# Supplementary material for: Prognostic and Therapeutic Potential of the OIP5 Network in Papillary Renal Cell Carcinoma
Source: Cancers (Basel). 2021 Sep 6;13(17):4483. doi: 10.3390/cancers13174483 (PMC8431695; doi:10.3390/cancers13174483)
Supplement: Supplementary file 1 [file cancers-13-04483-s001.zip › cancers-1344530-supplementary/Sup Fig S1.pdf]

Figure S1

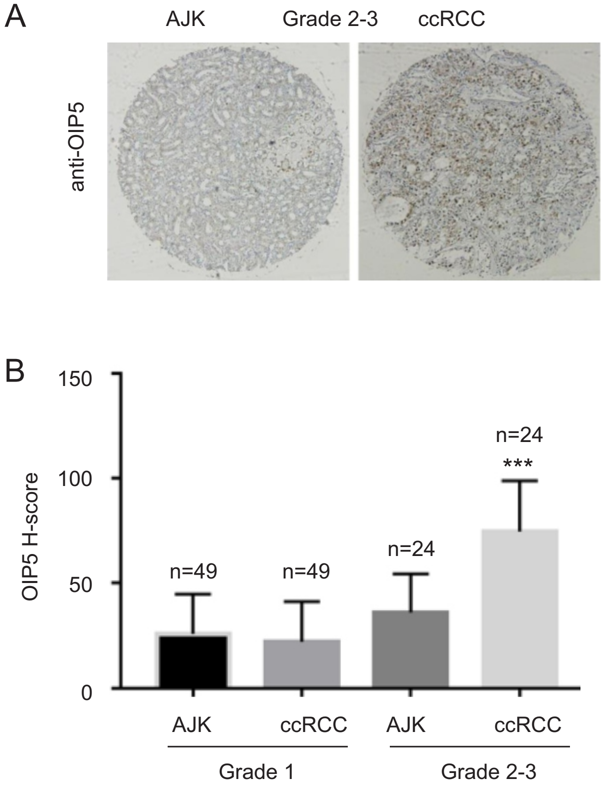

Figure S1. OIP5 upregulation in ccRCC. OIP5 expression was determined by IHC in a RCC tissue microarray and quantified using H-score. Typical images (**A**) and quantifications (mean ± SDs) (**B**) are presented. ***: $p < 0.001$ in comparison to the respective AJK tissues by 2-tailed Student t-test.
