# Supplementary material for: Prognostic and Therapeutic Potential of the OIP5 Network in Papillary Renal Cell Carcinoma
Source: Cancers (Basel). 2021 Sep 6;13(17):4483. doi: 10.3390/cancers13174483 (PMC8431695; doi:10.3390/cancers13174483)
Supplement: Supplementary file 1 [file cancers-13-04483-s001.zip › cancers-1344530-supplementary/Sup Fig S10 v1.pdf]

Figure S10

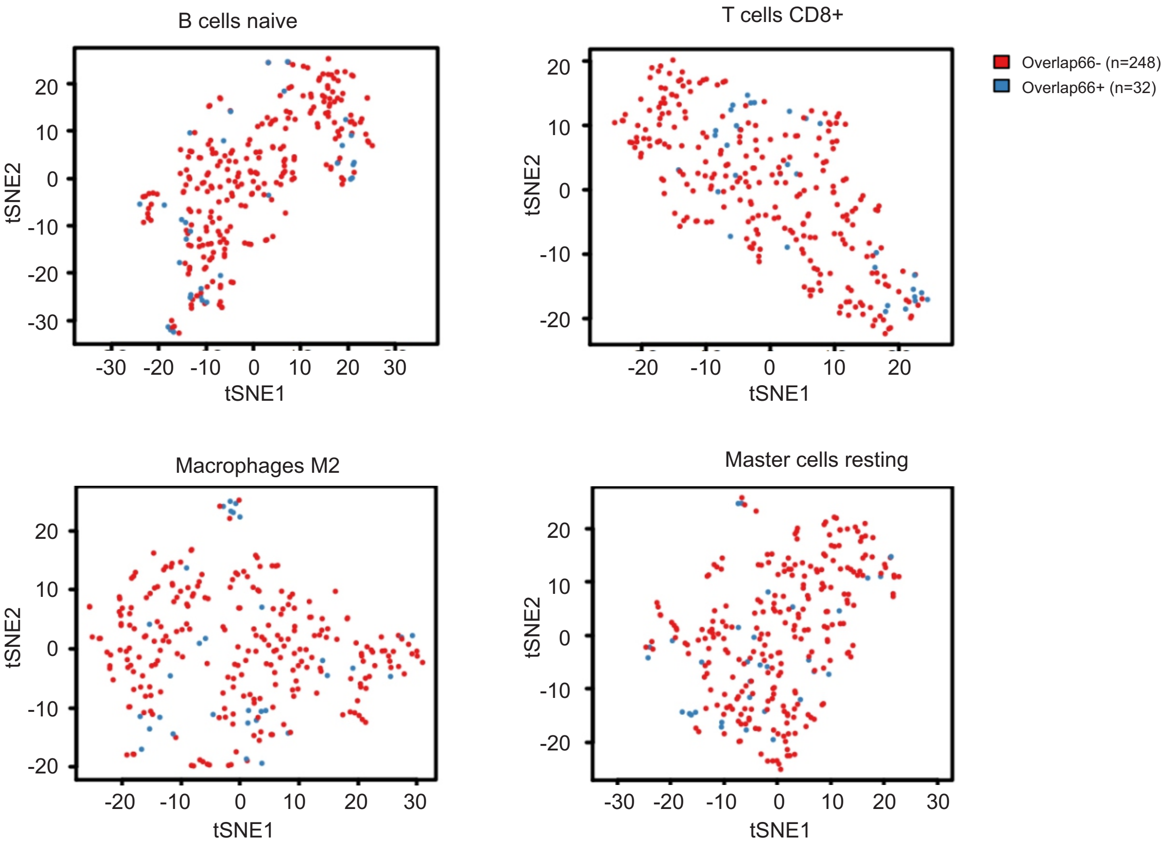

Figure S10. Clustering of the indicated immune cell subsets with tSNE. The graph was produced using the high-resolution mode within CIBERSORTx (https://cibersortx.stanford.edu/index.php) (see ref 47).
