# Supplementary material for: Prognostic and Therapeutic Potential of the OIP5 Network in Papillary Renal Cell Carcinoma
Source: Cancers (Basel). 2021 Sep 6;13(17):4483. doi: 10.3390/cancers13174483 (PMC8431695; doi:10.3390/cancers13174483)
Supplement: Supplementary file 1 [file cancers-13-04483-s001.zip › cancers-1344530-supplementary/Sup Fig S2.pdf]

Figure S2

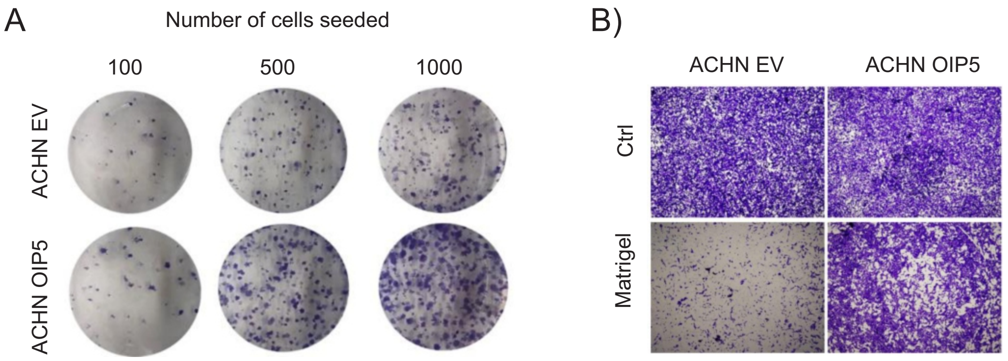

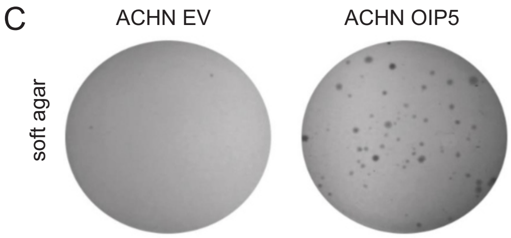

Figure S2. OIP5 enhances ACHN cell proliferation, colony formation, and growth in soft agar. **(A)** The indicated number of cells were seeded in a 6-well plate and cultured. Colonies were stained with crystal violet. **(B)** The indicated cells (10^4/well) were examined for invasion *in vitro* using a control (Ctrl) and Matrigel membrane. **(C)** Soft agar assay was conducted in 6-well plate.
