# Supplementary material for: Prognostic and Therapeutic Potential of the OIP5 Network in Papillary Renal Cell Carcinoma
Source: Cancers (Basel). 2021 Sep 6;13(17):4483. doi: 10.3390/cancers13174483 (PMC8431695; doi:10.3390/cancers13174483)
Supplement: Supplementary file 1 [file cancers-13-04483-s001.zip › cancers-1344530-supplementary/Sup Fig S3.pdf]

Figure S3

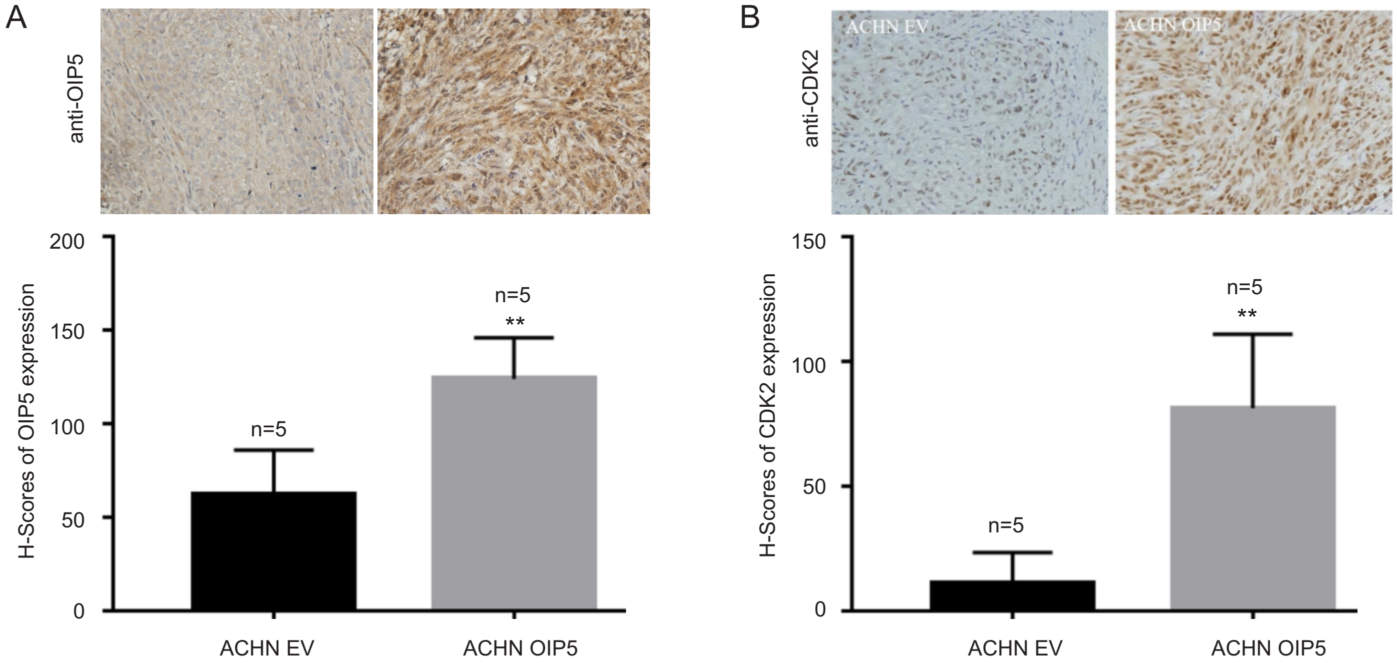

Figure S3. Increases in OIP5 and CDK2 expression in ACHN OIP5 tumors. Typical images for OIP5 (**A**) and CDK2 (**B**) expression and quantification. **: $p < 0.01$ compared ACHN EV tumors by 2-tailed Student's t-test.
