# Supplementary material for: Prognostic and Therapeutic Potential of the OIP5 Network in Papillary Renal Cell Carcinoma
Source: Cancers (Basel). 2021 Sep 6;13(17):4483. doi: 10.3390/cancers13174483 (PMC8431695; doi:10.3390/cancers13174483)
Supplement: Supplementary file 1 [file cancers-13-04483-s001.zip › cancers-1344530-supplementary/Sup Fig S4 v1.pdf]

Figure S4

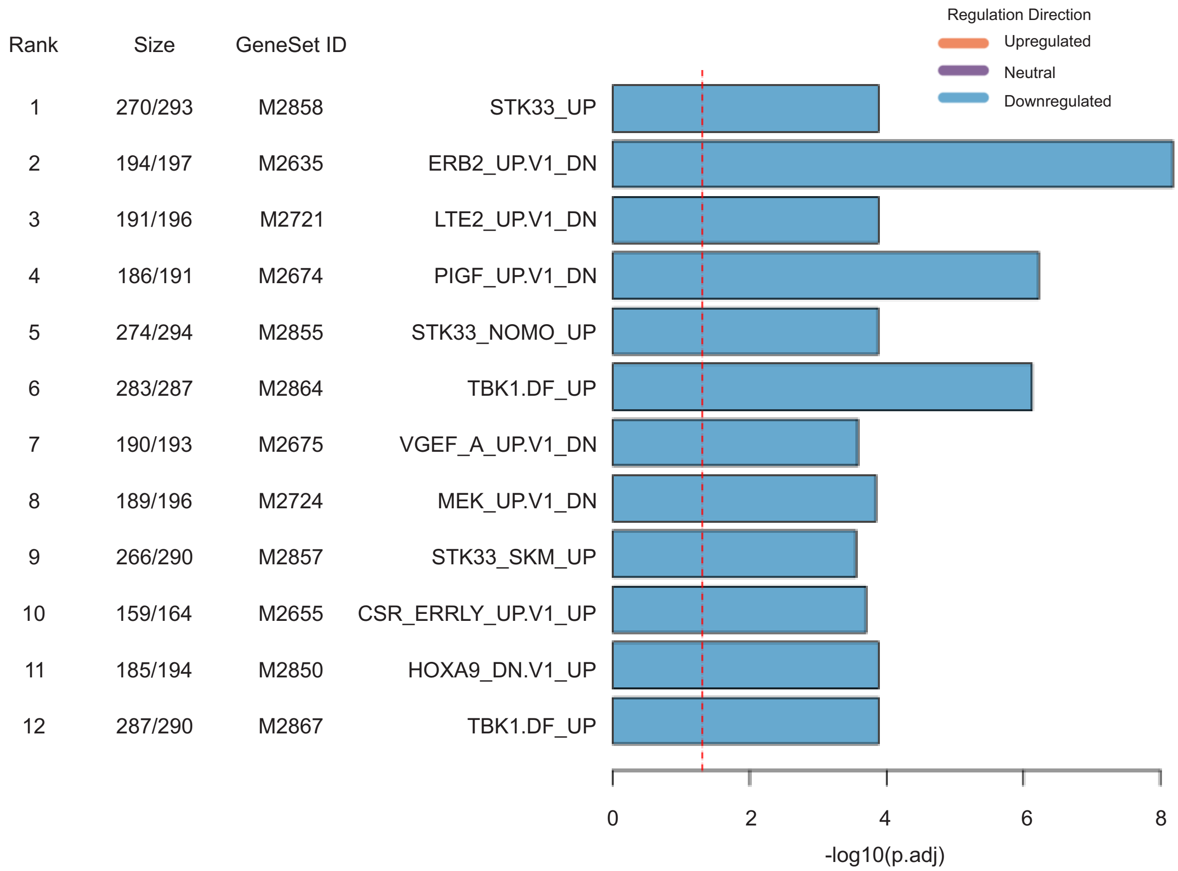

Figure S4. Gene set enrichment in ACHN OIP5 tumors. Gene set enrichment analysis was conducted using EGSEA (ensemble gene set enrichment test) within the Galaxy platform on differentially expressed genes obtained by comparison of ACHN OIP5 tumors (n=3) and ACHN EV tumors (n=3). The enrichment of the oncogenic (C6) gene set collection (MSigDB) was determined by a consensus gene set ranking among all 12 algorithms: *ora, globaltest, plage, safe, zscore, gage, ssgsea, padog, gsva, camera, roast,* and *fly.* Rank was sorted by median rank; size: number of genes in DEGs/number of genes in the gene set.

- STK33_UP: Genes up-regulated in NOMO-1 and SKM-1 cells after knockdown of STK33 by RNAi
- ERB2_UP.V1_DN: Genes down-regulated in MCF-7 cells engineered to express ligand-activatable ERBB2
- LTE2_UP.V1_DN: Genes down-regulated in MCF-7 cells and long-term adapted for estrogen-independent growth
- PIGF_UP.V1_UP: Genes up-regulated in HUVEC cells by treatment with PIGF
- STK33_NOMO_UP: Genes up-regulated in NOMO-1 cells (AML) after knockdown of STK33 by RNAi
- TBK1.DF_DN: Genes down-regulated in epithelial lung cancer cell lines upon over-expression of an oncogenic form of KRAS gene and knockdown of TBK1 gene by RNAi
- VEGF_A_UP.V1_DN: Genes down-regulated in HUVEC cells by treatment with VEGFA
- MEK_UP.V1_DN: Genes down-regulated in MCF-7 cells stably over-expressing constitutively active MAP2K1 gene
- STK33_SKM_UP: Genes up-regulated in SKM-1 cells after knockdown of STK33 by RNAi
- CSR_EARLY_UP.V1_UP: Genes up-regulated in early serum response of CRL 2091 cells (foreskin fibroblasts)
- HOXA9_DN.V1_UP: Genes up-regulated in MOLM-14 cells (AML) with knockdown of HOXA9 gene by RNAi vs controls
- TBK1.DF_UP: Genes up-regulated in epithelial lung cancer cell lines upon over-expression of an oncogenic form of KRAS gene and knockdown of TBK1 gene by RNAi
