# Supplementary material for: Prognostic and Therapeutic Potential of the OIP5 Network in Papillary Renal Cell Carcinoma
Source: Cancers (Basel). 2021 Sep 6;13(17):4483. doi: 10.3390/cancers13174483 (PMC8431695; doi:10.3390/cancers13174483)
Supplement: Supplementary file 1 [file cancers-13-04483-s001.zip › cancers-1344530-supplementary/Sup Fig S5 v1.pdf]

Figure S5

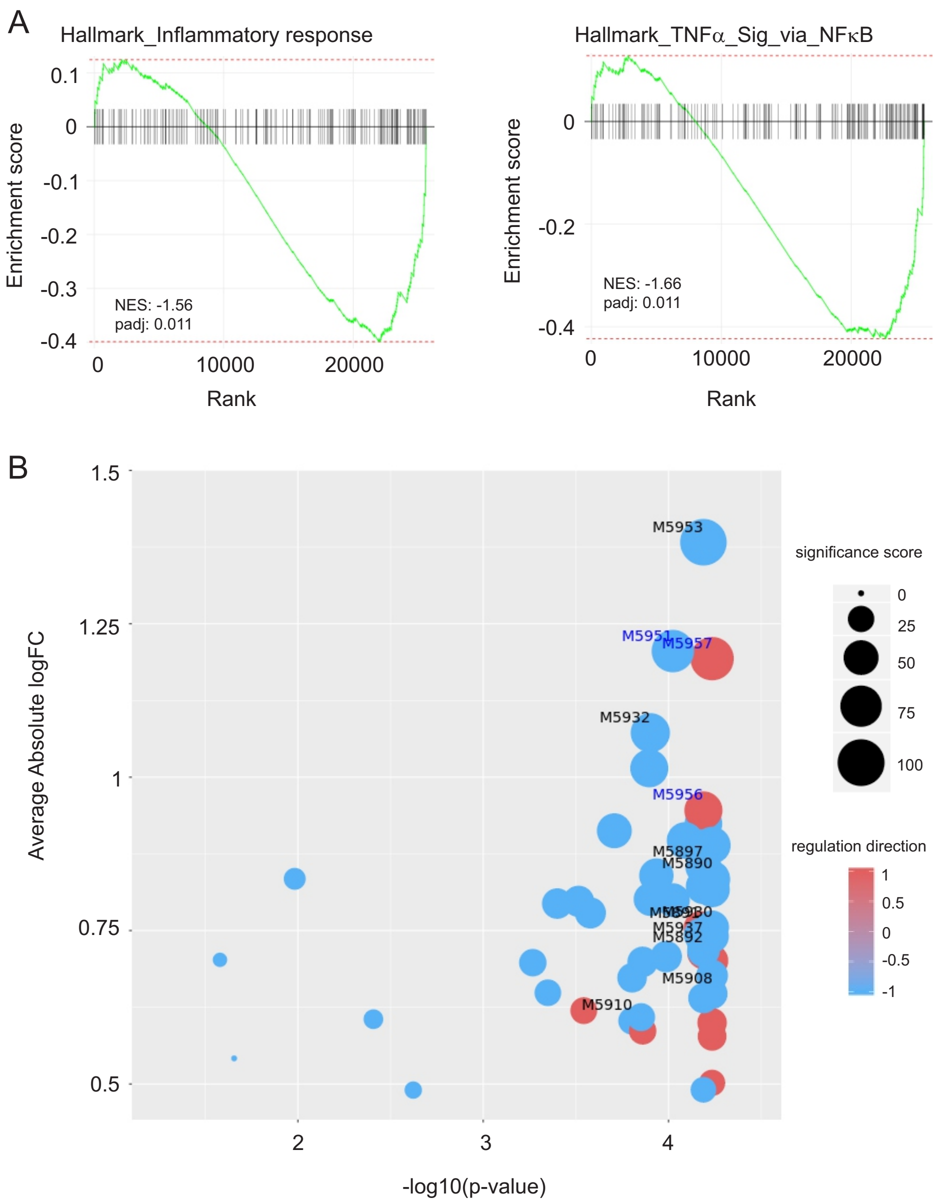

C

	ID	size	rank	p.adj	avg.logFC
Fatty acid metabolism	M5935	151/158	12	5.82e-05	0.60
Hypoxia	M5891	197/200	6	7.74e-05	0.754
Inflammatory response	M5932	185/200	8	1.25e-04	-1.072
TNFα_Sig_via_NFκB	M5890	192/200	7	5.82e-05	-0.835

Figure S5. Enrichment of h-Hallmarks Gene Set collection in ACHN OIP5 tumors. (**A**) Enrichment was analyzed using FGSEA within the Galaxy platform. **(B, C)** Enrichment analyses were performed using EGEA by UseGalaxy.org. The summary plot of mean absolute ford change (log2FC) and p.adj value (B). The details of the indicated gene sets, which were also derived from FGSEA analysis [panel A, Figure 3C], are presented (C).
