# Supplementary material for: Prognostic and Therapeutic Potential of the OIP5 Network in Papillary Renal Cell Carcinoma
Source: Cancers (Basel). 2021 Sep 6;13(17):4483. doi: 10.3390/cancers13174483 (PMC8431695; doi:10.3390/cancers13174483)
Supplement: Supplementary file 1 [file cancers-13-04483-s001.zip › cancers-1344530-supplementary/Sup Fig S6 v2.pdf]

Figure S6

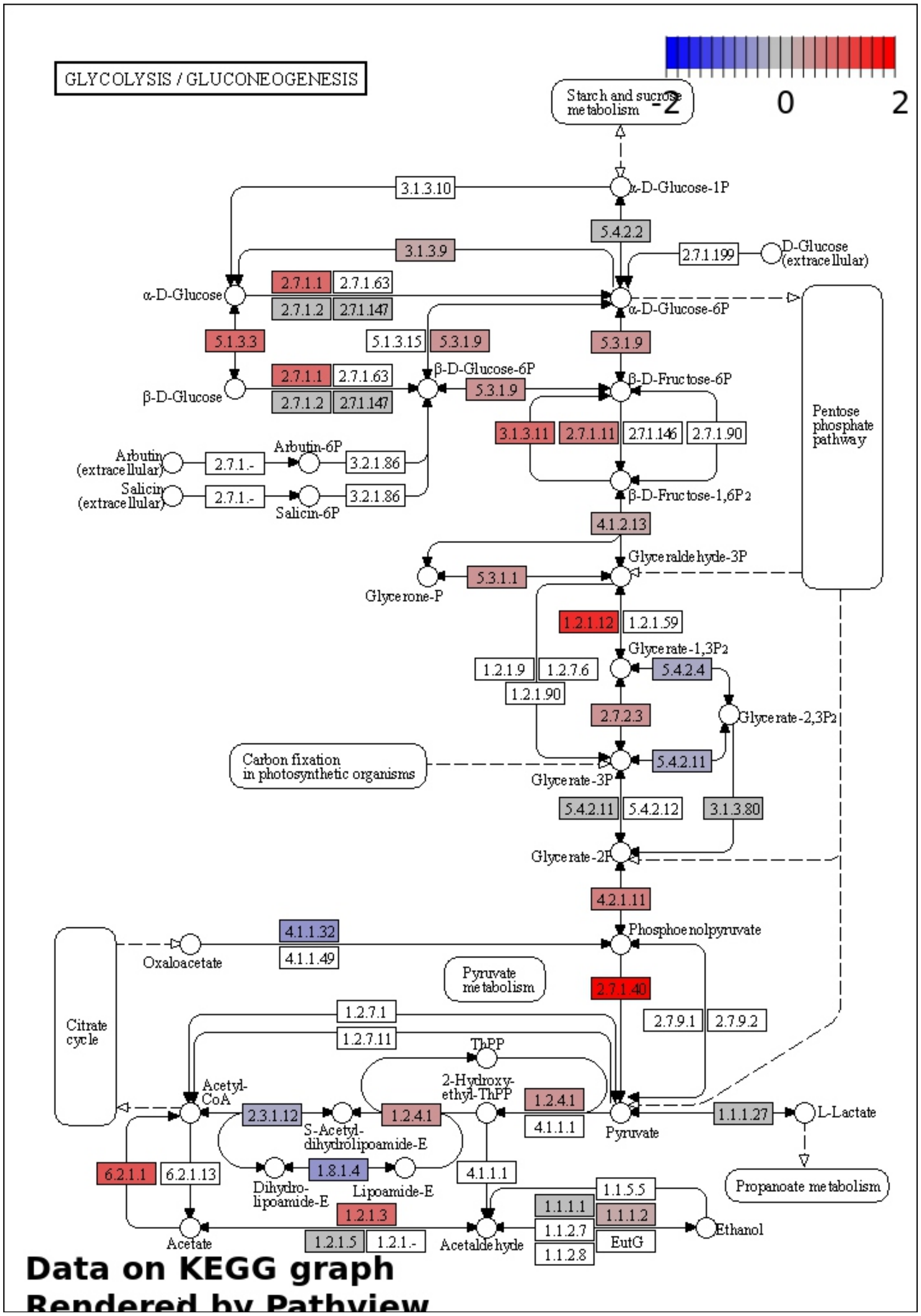

Rank: #3

Size: 62/67

Direction regulation: Upregulation

p.adj: 0.00356

Figure S6. Enhancement of the glycolysis pathway in ACHN OIP5 tumors. Pathway analysis was performed using EGSEA with the involvement all 12 algorithms by UseGalaxy.org.
