# Supplementary material for: Prognostic and Therapeutic Potential of the OIP5 Network in Papillary Renal Cell Carcinoma
Source: Cancers (Basel). 2021 Sep 6;13(17):4483. doi: 10.3390/cancers13174483 (PMC8431695; doi:10.3390/cancers13174483)
Supplement: Supplementary file 1 [file cancers-13-04483-s001.zip › cancers-1344530-supplementary/Sup Fig S7 v1.pdf]

Figure S7

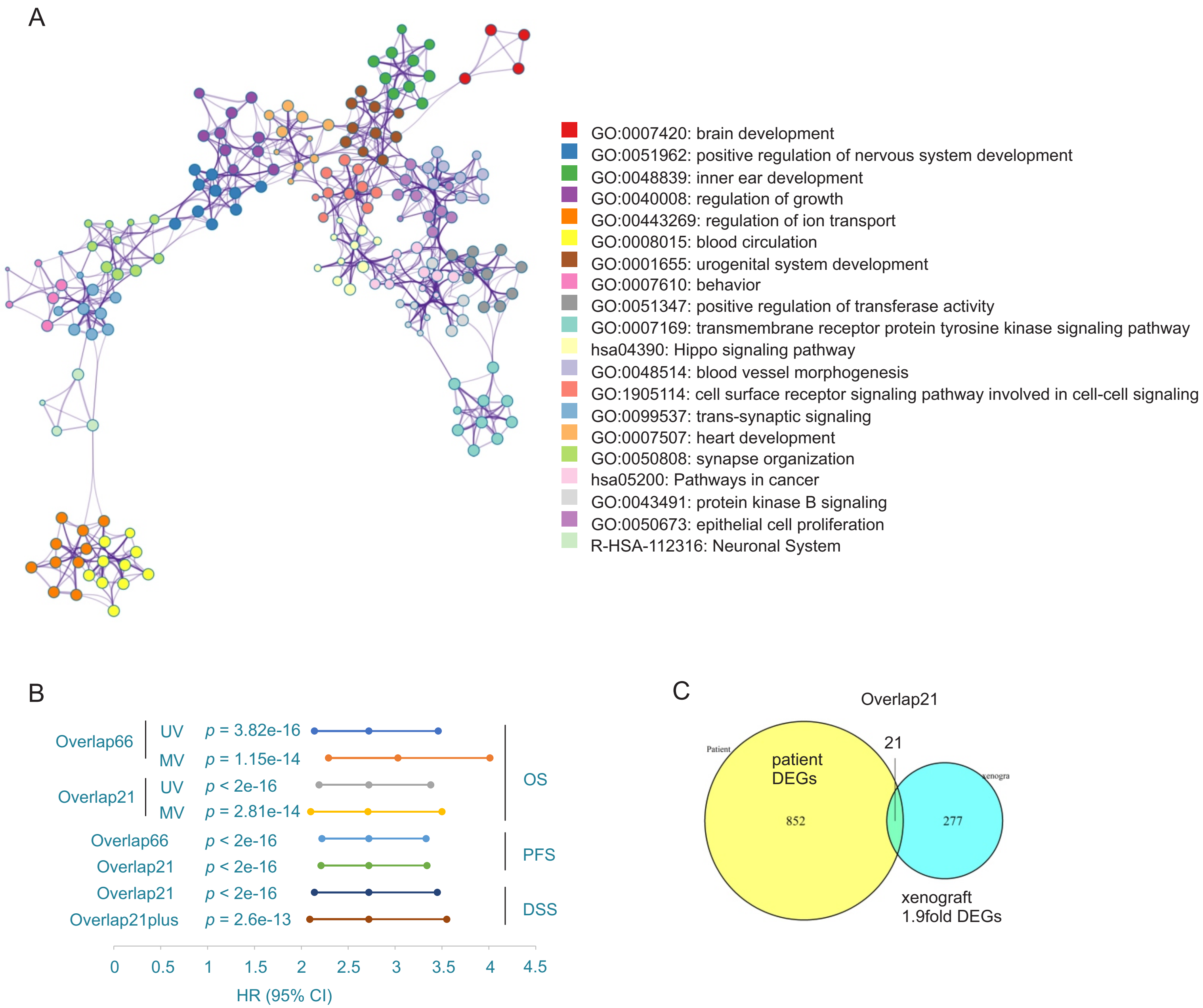

Figure S7. Prediction of OS, PFS, and DSS possibilities by Overlaop66, Overlap21, and Overlap21plus. **(A)** Network connection of individual enriched terms within individual clusters (see Fig. 4a, Additional file 10: Table S4 for details). **(B)** The predictions of OS, PFS, and DSS possibility using Overlap66, Overlap21, and Overlap21plus risk scores. UV and MV: univariate and multivariate Cox analysis, HR: hazard ratio, and CI: confidence interval. **(C)** The number of overlapping genes between primary (patient) pRCC-derived DEGs and DEGs obtained from xenografts at fold change ≥ |1.9|.
