# Supplementary material for: Prognostic and Therapeutic Potential of the OIP5 Network in Papillary Renal Cell Carcinoma
Source: Cancers (Basel). 2021 Sep 6;13(17):4483. doi: 10.3390/cancers13174483 (PMC8431695; doi:10.3390/cancers13174483)
Supplement: Supplementary file 1 [file cancers-13-04483-s001.zip › cancers-1344530-supplementary/Sup Fig S8.pdf]

Figure S8

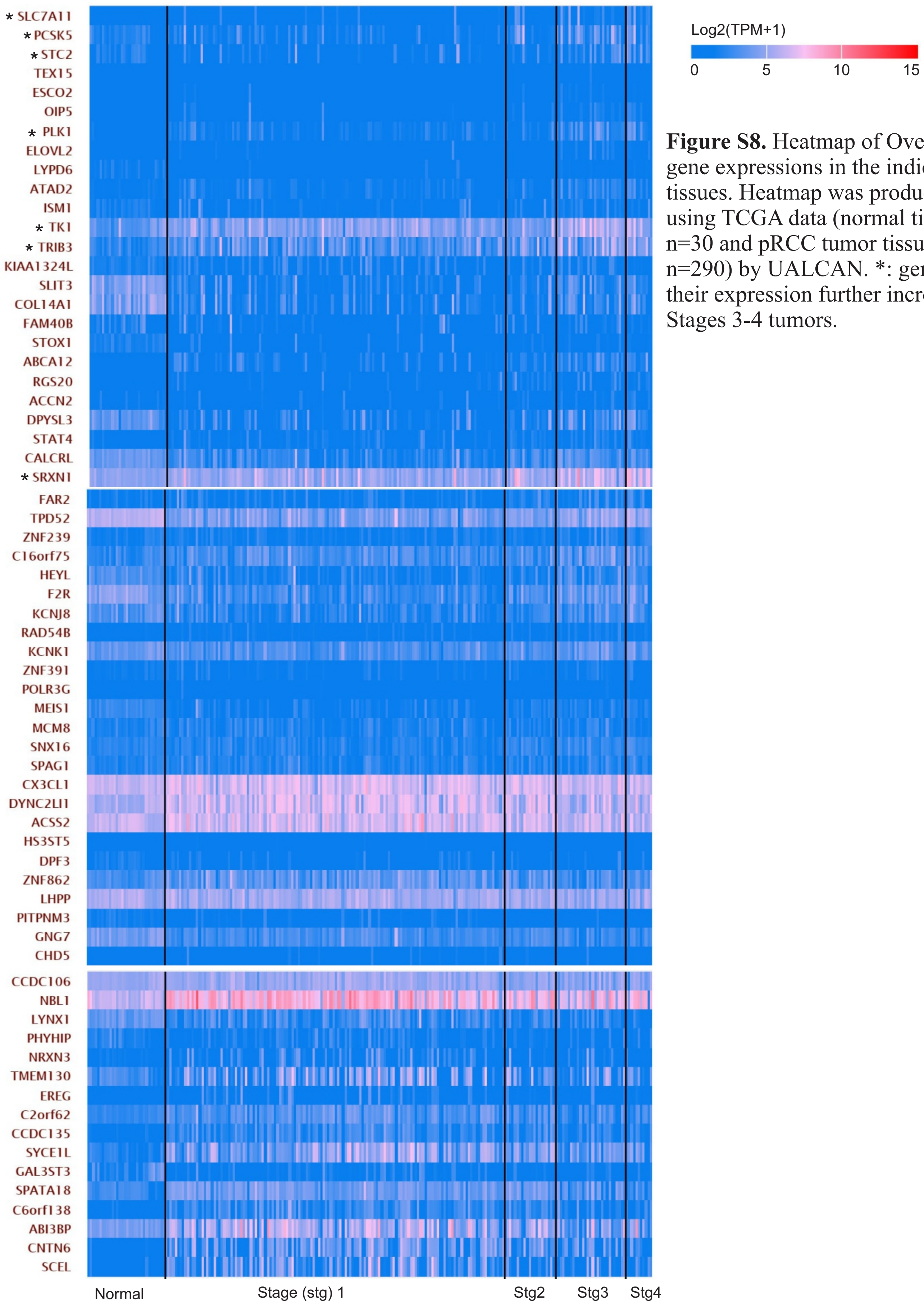

Figure S8. Heatmap of Overlap66 gene expressions in the indicated tissues. Heatmap was produced using TCGA data (normal tissues n=30 and pRCC tumor tissues n=290) by UALCAN. *: genes with their expression further increases in Stages 3-4 tumors.
