# Supplementary material for: Prognostic and Therapeutic Potential of the OIP5 Network in Papillary Renal Cell Carcinoma
Source: Cancers (Basel). 2021 Sep 6;13(17):4483. doi: 10.3390/cancers13174483 (PMC8431695; doi:10.3390/cancers13174483)
Supplement: Supplementary file 1 [file cancers-13-04483-s001.zip › cancers-1344530-supplementary/Sup Fig S9.pdf]

Figure S9

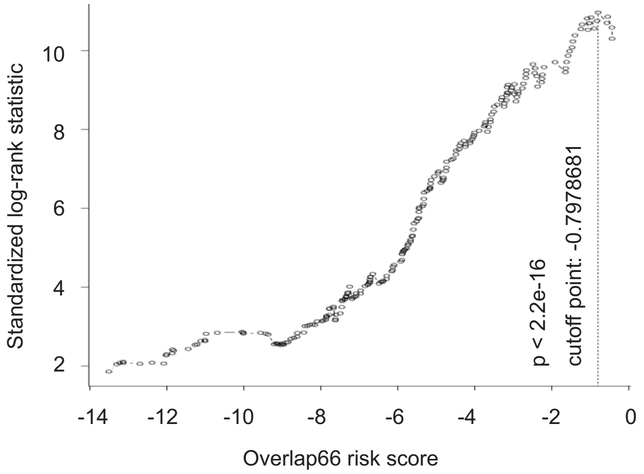

Figure S9. Cutoff point of Overlap66 risk score in the estimation of OS shortening. The cutoff point was determined using Maximally Selected Rank Statistics (the *Maxstat* package) in R.
