# Supplementary figures and images for: Prognostic and Therapeutic Potential of the OIP5 Network in Papillary Renal Cell Carcinoma
Source: Cancers (Basel). 2021 Sep 6;13(17):4483. doi: 10.3390/cancers13174483 (PMC8431695; doi:10.3390/cancers13174483)

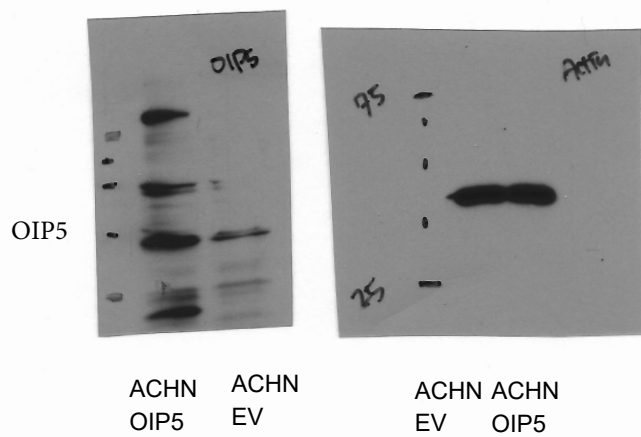

Supplement: Supplementary file 1 [file cancers-13-04483-s001.zip › cancers-1344530-supplementary/original WB figure.pdf]
